# Editorial: Autoantibodies and the role of RNA/RNA therapy in rheumatoid arthritis

**DOI:** 10.3389/fimmu.2022.1037843

**Published:** 2022-10-20

**Authors:** Kongyang Ma

**Affiliations:** Centre for Infection and Immunity Studies (CIIS), School of Medicine, Sun Yat-sen University, Shenzhen, China

**Keywords:** rheumatoid arthritis, scRNAseq, lncRNA, autoantibody, B cell

## Introduction

Rheumatoid arthritis (RA) is a systemic autoimmune disease characterized by the immune cell infiltration in the synovial joint. Autoantibodies have long been recognized as a hallmark of the development of RA in at least three ways. Firstly, rheumatoid factor and autoantibodies against post-translational modified proteins like citrullination (ACPA) have been used as diagnostic markers in RA. Secondly, the broad cross-reactivity of ACPA may contribute to novel target antigen definition in RA (1). Thirdly, several pathogenic B cell subsets, such as PD-1^+^ B cells, CD27^+^IgD^-^ memory B cells, and B-1a cells may participate in RA pathogenesis and treatment directly or indirectly *via* autoantibody secretion (2, 3). Therefore, the presence of autoantibodies can be useful in RA prediction, diagnosis, and treatment.

Due to the progressive joint destruction in RA patients, novel strategies including RNA therapy are eagerly needed. As editors for the Research Topic, we review excellent articles within this field. We summarize the main contributions and perspective clues of the accepted articles in this editorial.

## Mechanism of autoantibody in RA

The presence of anti-carbamylated protein autoantibodies (anti-CarP) is a hallmark of RA and is associated with bone erosion (4). O’Neil et al. conducted immune precipitation IP and ELISA assay, aiming to identify novel carbamylated antigens in patients with RA. They found the significant elevation of carbamylated LL37 (carLL37) in sera and synovial fluid from RA patients using the ELISA assay, since LL37 could be internalized during neutrophil extracellular trap (NET) formation (5). The persistence of carLL37 was also confirmed by the co-IP of NETs from RA patients, indicating that the carLL37–NET complex contributes to the autoantigen pool during RA pathogenesis.

A mechanism study used carLL37–NET-treated RA fibroblast-like synoviocytes (FLSs) and observed the internalization of carLL37 probably by the MHCII compartment. In humanized HLA-DRB4*04:01 transgenic mice, O’Neil et al. used repeated immunization with carLL37–NET-treated FLSs and observed a significant increase in anti-carLL37 antibody generation. Importantly, the elevated levels of anti-carLL37 autoantibodies were detected in the RA synovium and positively correlated with joint erosion. An *in vitro* culture system showed that the treatment of carLL37–IgG immune complexes could promote osteoclast formation. O’Neil et al. revealed that the pathogenic roles of dysregulated NET formation and the released car-LL37 triggered autoimmune response during joint damage in RA, leading to the novel therapeutic interventions of RA treatment (5).

## Mechanism of B cells in RA

As the source of autoantibodies and cytokines including RANKL (2, 3), B cell targeted therapy has well proved its importance during RA pathogenesis (6). Active RA B cells especially in the synovial ectopic lymphoid structures (ELSs) are of great interest (7). Wu et al. reviewed the abnormal immune checkpoint signals of RA B cells, e.g., BCR, TLR, CD40, BAFF, APRIL, IL-21, and IL-6. They also summarized the multiple functions of RA B cells, such as antigen presentation, cytokine production, and autoantibody secretion, and the prospective B cell therapies targeting B cell surface receptors and checkpoints, such as CD20, CD38, BAFF-R, TACI, BCMA, CD40, and so on. Currently, accumulating evidence supports the pathogenic roles of B cells during RA development and joint damage, and more interventions on inhibiting the overactivation and eliminating the expansion of pathogenic B cell subsets will be explored further.

## Mechanism of LncRNAs in RA

The role of long non-coding RNAs (LncRNAs) has been implicated in RA (8). Huang et al. summarized four types of LncRNAs with distinct functions, i.e., the signal, decoy, guide, and scaffold LncRNAs. They listed the key LncRNAs in modulating the inflammatory cytokine secretion of FLSs, controlling the polarization and differentiation of T cells and macrophages, and modifying the autoantibody production of B cells. Briefly, the upregulated Lnc00152 could activate the TGF-β-activated kinase 1 (TAK1)-mediated NF-κB signaling and promote TNF-α secretion by targeting miR-103a (9). Moreover, the downregulation of LncRNA GAS5 in RA FLS promoted the TNF-α secretion in RA FLS as well (10). The upregulation of LncRNA IFNG-AS1 in RA patients enhanced the transcription of IFN-γ-encoding genes during Th1 differentiation. Especially, the TT genotype of rs2067079 single-nucleotide polymorphism (SNP) in LncRNA GAS5 was associated with a significantly decreased risk of RA (11).

Recently, the siRNA technology, by targeting the RA-related LncRNAs, has been proven to inhibit inflammatory response and joint damage. Thus, a further understanding of LncRNAs in RA pathogenesis is critical for developing new therapeutic strategies in clinic.

## Application of scRNAseq in RA

Single-cell RNA sequencing (scRNAseq) is severe as a powerful tool for interrogating rheumatic diseases (12, 13). In the review of current single-cell investigations in autoimmune rheumatic diseases, Zheng et al. summarized the cutting-edge research on the elucidation of the cellular atlas including novel cell populations and the pathogenic transcriptome signature of various cell types in RA. Patient sample collections from the peripheral blood and inflamed tissue helped measure the phenotypic divergence of novel cell populations with distinct functions and their contribution to disease manifestations, for instance, the discovery of synovial local THY1(CD90)^+^ HLA–DRA^hi^ FLSs with key chemokine expression signatures, IL1β^+^ pro-inflammatory monocytes, and ITGAX^+^ TBX21^+^ autoimmune B cells by integrating single-cell transcriptomics and mass cytometry (14). Importantly, the scRNAseq analysis in stromal cells, such as Thy1^+^ FLSs, may provide the perspective clues for local treatment in RA patients.

Moreover, the concurrent TCR and/or BCR sequencing of these samples would also be immensely helpful to track the clonal lineage of lymphocyte populations and potentially track the differentiation trajectory followed by tissue-infiltrating cells. Although the increased resources of scRNAseq data in RA patients greatly supported the investigation of novel transcriptome features and novel transcription factors, more validation in protein levels and cell function is important for the further application of scRNAseq data in research and clinic.

## Perspectives

In conclusion, this Research Topic provided multiple aspects of views in the pathogenesis, diagnosis, and clinical intervention of RA, focusing on the autoantibodies and RNA/RNA therapies in RA. Raising strategies have been developed in the treatment of RA; nevertheless, there remained huge challenges in the mechanism studies and RNA therapy of RA.

## Author contributions

The author confirms being the sole contributor of this work and has approved it for publication.

## Funding

This study was supported by the National Natural Science Foundation of China (No. 82171782; No. 81901635).

## Acknowledgments

We appreciate the contributions of all the authors and reviewers who have participated in this Research Topic.

## Conflict of interest

The author declares that the research was conducted in the absence of any commercial or financial relationships that could be construed as a potential conflict of interest.

The reviewer FX declared a shared affiliation with the author the handling editor at the time of review.

## Publisher’s note

All claims expressed in this article are solely those of the authors and do not necessarily represent those of their affiliated organizations, or those of the publisher, the editors and the reviewers. Any product that may be evaluated in this article, or claim that may be made by its manufacturer, is not guaranteed or endorsed by the publisher.
